# Guiding prevention initiatives by applying network analysis to systems maps of adverse childhood experiences and adolescent suicide

**DOI:** 10.1017/nws.2024.8

**Published:** 2024

**Authors:** Benjamin D. Maldonado, Ryan Schuerkamp, Cassidy M. Martin, Ketra L. Rice, Nisha Nataraj, Margaret M. Brown, Christopher R. Harper, Curtis Florence, Philippe J. Giabbanelli

**Affiliations:** 1 Department of Computer Science and Software Engineering, Miami University, Oxford, OH, USA; 2 National Center for Injury Prevention and Control, Centers for Disease Control and Prevention, Atlanta, GA, USA; 3 Defense Suicide Prevention Office, Department of Defense, Washington, DC, USA

**Keywords:** Adverse childhood experiences, adolescent suicide, causal map, node centrality, community detection, network analysis, suicide prevention

## Abstract

Suicide is a leading cause of death in the United States, particularly among adolescents. In recent years, suicidal ideation, attempts, and fatalities have increased. Systems maps can effectively represent complex issues such as suicide, thus providing decision-support tools for policymakers to identify and evaluate interventions. While network science has served to examine systems maps in fields such as obesity, there is limited research at the intersection of suicidology and network science. In this paper, we apply network science to a large causal map of adverse childhood experiences (ACEs) and suicide to address this gap. The National Center for Injury Prevention and Control (NCIPC) within the Centers for Disease Control and Prevention recently created a causal map that encapsulates ACEs and adolescent suicide in 361 concept nodes and 946 directed relationships. In this study, we examine this map and three similar models through three related questions: (Q1) how do existing network-based models of suicide differ in terms of node- and network-level characteristics? (Q2) Using the NCIPC model as a unifying framework, how do current suicide intervention strategies align with prevailing theories of suicide? (Q3) How can the use of network science on the NCIPC model guide suicide interventions?

## 1. Introduction

Suicide is the second leading cause of death among adolescents ages 10–14 and third leading cause of death among adolescents ages 15–19 in the United States, accounting for 17.0% of all deaths among those aged 10–14 and 18.0% of all deaths among those aged 15–19 (Centers for Disease Control and Prevention’s National Center for Injury Prevention and Control, 2020). Moreover, suicide rates among adolescents are on the rise: between 2009 and 2018, suicide rates among high school-aged children (i.e., ages ranging from 14 to 18) increased by 61.3% (Ivey-Stephenson et al., 2020). Suicide attempts have likewise increased, doubling in a similar period between 2008 and 2015. Within that same high school age category, 18.8% of students reported seriously considering suicide, and 15.7% reported making a suicide plan. With suicide attempts and *suicidal ideation* (“a broad term used to describe a range of contemplations, wishes, and preoccupations with death and suicide” (Harmer et al., 2020)) on the rise in recent decades, a public health solution is needed to effectively address the complex causes of suicidal behaviors.

Prior research has developed several models to capture the inherent complexity behind suicidal ideations (Brenas et al., 2019; Chung, 2016; Page et al., 2018). Although these models use different approaches (e.g., ontologies, System Dynamics), they are *all structured as networks* consisting of concepts for nodes (e.g., homelessness, depression) and directed edges for the impact of one concept upon another. These models recognize suicide as a multifactorial problem, thus using networks to represent how suicide is shaped by (and contributes to) multiple causes instead of having a single “root” cause. As such networks are comprehensive, they can consist of a large number of factors and interrelationships. Visualizing all the content at once can be overwhelming for users and emphasizes the complexity of a problem, instead of focusing on specific solutions. Consequently, such network-based models are primarily used as decision-support systems either to understand the dynamics of a problem (e.g., by identifying key themes) or by examining the potential effects of interventions. Recently, researchers at the National Center for Injury Prevention and Control (NCIPC) within the CDC created a new causal map regarding the complexities linking Adverse Childhood Experiences (ACEs) to suicide ideation, attempt, and fatality among youth. The map was obtained by a participatory modeling project involving fifteen subject matter experts (SMEs) on various facets of ACEs and suicide (e.g., epidemiologists, psychiatrists, psychologists). We refer to this causal map as “the NCIPC model,” and its content is briefly summarized in the Appendix. It captures adolescent suicide through 361 nodes and 946 edges (Giabbanelli et al., 2021), which makes it a sizable systems map to both understand the problem and guide prevention initiatives.

The release of a map following a participatory modeling initiative is usually followed by analytical efforts rooted in network science. The Foresight Obesity Map (McPherson et al., 2007) illustrates this approach: after its release in 2007, practitioners observed that the network was too large to be simply “looked at” (Siokou et al., 2014), hence network analyses were conducted to find useful patterns for obesity prevention. Such analyses have included the *identification of communities*, revealing themes that could centralize prevention efforts (Finegood et al., 2010; Drasic and Giabbanelli, 2015) (e.g., body image, physical well-being). Node centrality has also been a common analysis, particularly with regard to finding leverage points to intervene in a system (McGlashan et al., 2016). The Foresight Obesity Map has also been compared with another map, using themes and node centrality to contrast the focal points of these two maps and their implications for obesity prevention initiatives (McGlashan et al., 2018). The field of obesity is well-known for taking a “whole-system approach” resulting in an abundance of maps and *comparative network studies*, and other fields such as diabetes (Giles et al., 2007) and socio-environmental management (Lavin et al., 2018) have also performed such analyses. Despite a recent growth of modeling studies applied to suicide (Schuerkamp et al., 2023), fewer systems maps are devoted to suicide and no comparative network study has taken place into this context yet. In this paper, we perform the first comparative analyses through four such models for suicide and ACEs: two models with a focus on suicide (Chung, 2016; Page et al., 2018), one emphasizing ACEs (Brenas et al., 2019), and the NCIPC model touching on both aspects.

Our study addresses three questions:

Q1: What are the differences between network-based models for suicide prevention? We address this question by representing the four detailed models above-mentioned as a network. Following established practices in terms of contrasting themes and central nodes, we assess each network with respect to both overall structure (density, diameter) and key factors (via five measures of node centrality).
Q2: Using a network model as a unifying framework, how are current suicide intervention packages aligned with prevailing theories for suicide? This question cannot be addressed solely based on the network, as we need to contextualize its content *with respect to theories*. We thus use a mixed-methods approach by examining network elements through a policy lens. Specifically, we categorize each node with respect to both the CDC suicide prevention technical package of policy, programs, and practices and the Social-Ecological Model (SEM) Framework (Stone et al., 2017; Centers for Disease Control and Prevention’s National Center for Injury Prevention and Control, 2021).
Q3: How can a network model guide suicide interventions? While Q1 emphasizes the comparison of structural elements among models, Q3 focuses on the NCIPC model since it was recently released with the aim of guiding the design of suicide prevention interventions. In Q3, our analysis complements a purely static perspective (“what is in the model”) with a dynamic approach, since interventions consist of *what-if* questions that require investigating potential pathways and rippling effects. We thus use algorithms to identify the major systems components (i.e., community detection) and demonstrate how to perform intervention-oriented tasks (e.g., identifying unintended consequences).

The remainder of this paper is organized as follows. In Section 2, we describe systems maps (including causal maps, stock and flow diagrams, and ontologies) together with the process for their creation and analysis. Section 3 details the mixed methods used to address the three questions above, with a subsection dedicated to each question. Similarly, our results in Section 4 are organized to provide quantitative and qualitative answers to each question. In Section 5, we discuss some of the limitations of this study inherent to the field of suicide prevention. Section 6 summarizes our main contributions at the interface of applied network science and suicide.

## 2. Background: systems maps

### 2.1 What is a systems map?

System mapping is a tool originating from systems thinking in which a given domain is represented as a set of components (nodes) and relationships (edges). While components always have a name (i.e., node labels), the type of edges depends on the specific representation of a system used in a given project. One such representation is a *causal map*, in which categorized edges labeled positive (+) or negative (−) link various quantifiable factors to create a directed network (Firmansyah et al., 2019). A *positive edge* from one factor to another implies positive causation (an increase in the first factor will cause an increase in the second), while a *negative edge* implies negative causation (an increase in the first factor will cause a decrease in the second). For example, a positive edge from the node representing suicide ideation to the node representing suicide attempts means that an increase in suicide ideation leads to an increase in suicide attempts. Other representations of a system include stock and flow diagrams used for System Dynamics, where an edge may have a numerical value that is encoded within a modeling software rather than displayed on a schema. Ontologies are a related representation, in which the meaning and characteristics of a system can be conveyed in more details by equipping concepts with properties or associating them with entries in a glossary.

Several features present in complex problems such as *feedback loops*^1^ (when a change in one factor ultimately affects itself) and factors with a high number of outgoing relationships can be represented in systems maps (Giabbanelli et al., 2021). As a result, these maps are a powerful tool to analyze complex problems.

### 2.2 Why make a systems map?

Mapping a preexisting system is now one of the key stages in identifying and evaluating interventions for public health programs (Luna Pinzon et al., 2022). Creating and visualizing a systems map can exhibit the intricacies of the issue at hand and highlight the interdependence of factors through feedback loops (Finegood et al., 2010). Depicting feedback loops is critical as it helps decision-makers to comprehend the relationships between variables and begin to weigh the ramifications of policies and interventions. *Interventions* are actions taken to change variables in a system to achieve a specific outcome—e.g., reducing access to lethal means among persons at risk of suicide (Stone et al., 2017). Identifying feedback loops also empowers decision-makers to utilize naturally occurring system dynamics in their interventions (Firmansyah et al., 2019). For example, implementing social-emotional learning programs in schools may increase coping and problem-solving skills, which may, in turn, reduce suicide attempts (Stone et al., 2017).

System maps also allow users to identify a group of factors, the variables affected by them (both directly and indirectly), and the nature of these effects (e.g., by monitoring their type in a causal map) (Firmansyah et al., 2019). Without a systems map, tracing the effects of variables throughout the system would likely be more difficult. The process of creating a systems map can have widespread benefits even if the map is not widely used. Bringing together a broad range of stakeholders to form the map can help “forge multisector, multidisciplinary relationships that support future decision-making based on evidence” (Finegood et al., 2010). Moreover, stakeholder participation in creating a map can increase the legitimacy of decisions (Firmansyah et al., 2019). Combining both factors can help boost the quality and support of decision-making.

### 2.3 How to create a systems map?

**Figure 1.**
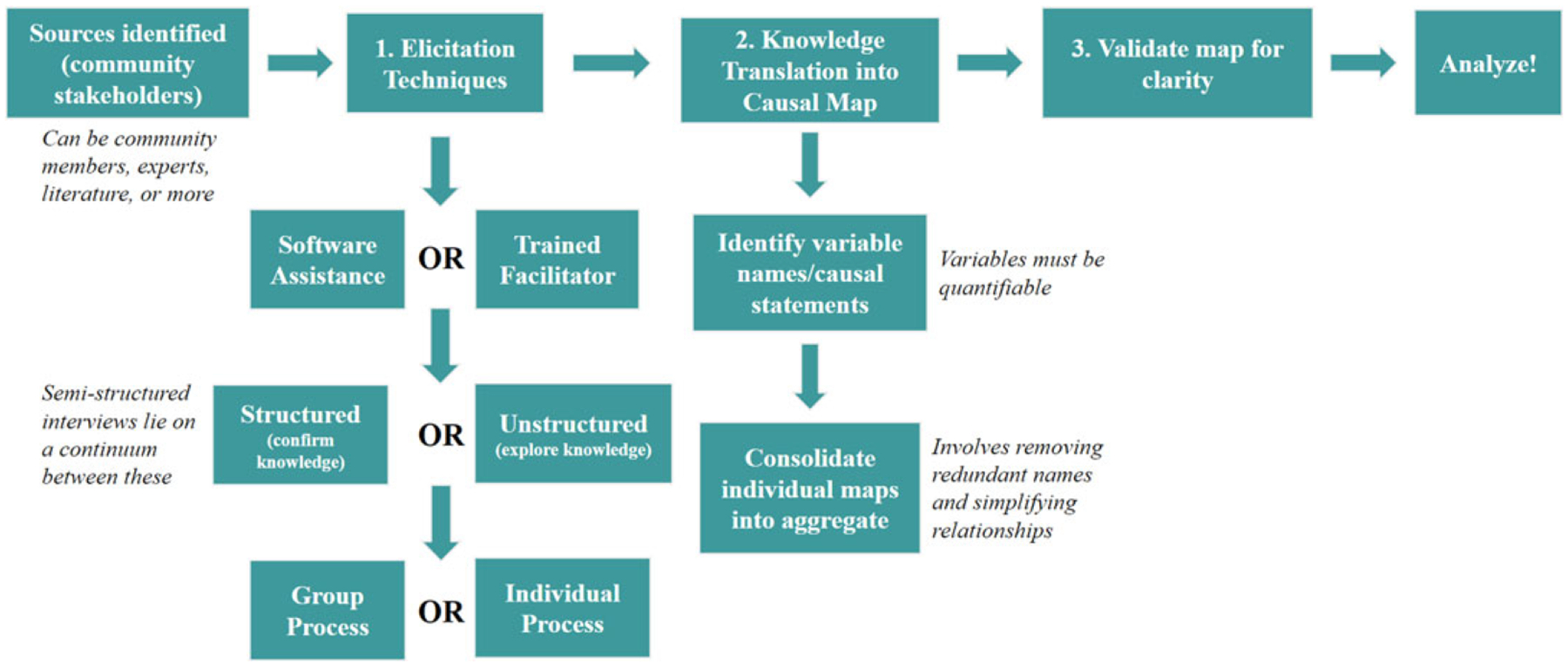
General process for creating a systems map.

The general process of constructing systems maps with participants has recently been covered in several articles, with respect to either knowledge representation (Giabbanelli and MacEwan, 2024) or the interactions between facilitators and participants (Knox et al., 2024). We thus provide a more succinct overview here, based on the diagram in Figure 1. One approach to create a map is to engage in participatory modeling. In this case, modelers perform *community-based participatory research* (CBPR) by building a collaborative partnership with community members through participatory activities (McGlashan et al., 2019). A model is created in CBPR by identifying and recruiting eligible participants, then obtaining informed consent. Participants are selected based on the problem boundaries; for instance, in the case of the model on adolescent suicide, individuals whose experience lies entirely on suicide in adulthood would not be engaged (Giabbanelli et al., 2021). Although research areas such as educational technology tend to use software so that each individual directly creates their map (e.g., cMap, jMap, Coggle), CBPR has fewer platforms (e.g., STICKE (Hayward et al., 2020)) and tends to employ trained facilitators who will support participants in developing maps. Data elicitation techniques can either be performed with a group, which will automatically result in a group-level model or with individuals, whose models are later combined into a group-level model (Giabbanelli et al., 2021).

When working with individuals, information is elicited using *unstructured techniques*, which rely on asking open-ended questions to externalize the participants” perspectives without imposing the bias of the interviewer on the interviewee. Transcribed interviews can be analyzed to identify variable names and the nature of their relationships, represented as nodes and edges, respectively. When building a causal map, relationships encode the *direction* and *type* of impact. For example, “traumatic experiences increase the risk for suicide ideation” and “having meaningful relationships with positive peers protects against suicide attempts.” Consequently, causal maps use directed, typed edges. In the two examples, the statements could be represented as *traumatic experiences $\overset{+}{\to }$ suicide ideation* and *connectedness $\bar{\to }$ suicide attempts*, respectively. In the case of ontologies or stock and flow diagrams, relationships would have attributes or numerical values, respectively.

Even when they share the same interest, modeling projects may differ in some of the sub-steps (Figure 1) based on considerations such as experience and availability of the facilitator (e.g., do they prefer one-on-one sessions or group workshops?), access to participants (e.g., can they all come to the same room?), or time constraints (e.g., do we need to produce a map by the end of the workshop?). The NCIPC model employed one-on-one interviews with a trained facilitator and an observer, then a causal map was produced from each interview (including typed and directed edges), and maps were aggregated with the assistance of SMEs to identify semantically equivalent constructs (Giabbanelli et al., 2022). The NCIPC model thus only reflects the views of the participants involved. The stock and flow diagram produced by Page and colleagues involved workshops and a wide representation “from health and social policy agencies, local councils, non-government organizations, emergency services, primary care providers, program planners, research institutions, community groups and those with lived experience of suicide” (Page et al., 2018). The model-building method thus differs from the NCIPC case, which relied solely on experts (e.g., epidemiologists, psychologists). As is commonly the case, the model by Page et al. was developed using the same protocols that the team applied for CBPR research in other domains, such as diabetes management (Freebairn et al., 2016) and alcohol-related harms (Atkinson et al., 2017). The model by Page et al. proceeds in a series of steps, from the general population to vulnerable individuals, who can become distressed and attempt suicide, leading either to fatality or post-attempt treatment.

CBPR is not the only way to create a map. For example, participants may be unavailable for a research team, or the modelers may consider that the evidence-base is sufficient to derive a map. The stock and flow model created by Chung thus *indirectly* builds on the work of experts, by expanding on the Interpersonal Theory of Suicide (Chu et al., 2017). This theory posits that suicide ideation happens when individuals feel that they are a burden and do not belong (i.e., the core constructs of belongingness and burdensomeness). In addition, the theory considers that attempts are enabled when individuals have a desire for suicide and no longer fear death (i.e., capability) (Vélez–Grau et al., 2023; Kelley and DeShong, 2023). Chung created the model by building on theory and identified specific variables using data from the In-Home surveys administered as part of AddHealth, a widely studied dataset created by experts. As a result, the model includes constructs such as depression (characterized by e.g., average duration and depression level), perceived self-worth, desire to die by suicide, fear of death (which is reduced by attempts and exposure to painful experiences), hopelessness, burdensomeness, and cultural stigma associated with suicide. The ontology by Brenas et al. (Brenas et al., 2019) also built on prior work, as it (i) reused the seminal study of Felitti and colleagues to identify relationships between Adverse Childhood Experiences (Felitti et al., 1998), (ii) reused medical ontologies to cover concepts such as abuse or mental illnesses, and (iii) connected the concepts as needed for the purpose of their study. The constructs thus cover mental health, physical harm (detailed as being hit, push, from thrown object, etc.), substance use and abuse, housing problems (e.g., low-quality housing or homelessness), and their effects on health (detailed via e.g., exposure to bug infestation, lead-based paint, mold, water leaks), triggering events (e.g., death, incarceration, parental separation or divorce), and access to care (e.g., transportation).

### 2.4 Analyzing systems maps

In a comparative network analysis, we need to assess the models based on *shared features*. Regardless of whether they originated as ontologies, causal maps, or stock and flow diagrams, all four models share a representation as directed, labeled networks. However, edges have different information, such as types (+ and − in causal maps), numerical weights or rates per time unit (stock and flow diagrams), or properties (ontologies). We can thus analyze all four models by accounting for the direction of their edges, but not in terms of edge weights or edge types.

**Figure 2.**
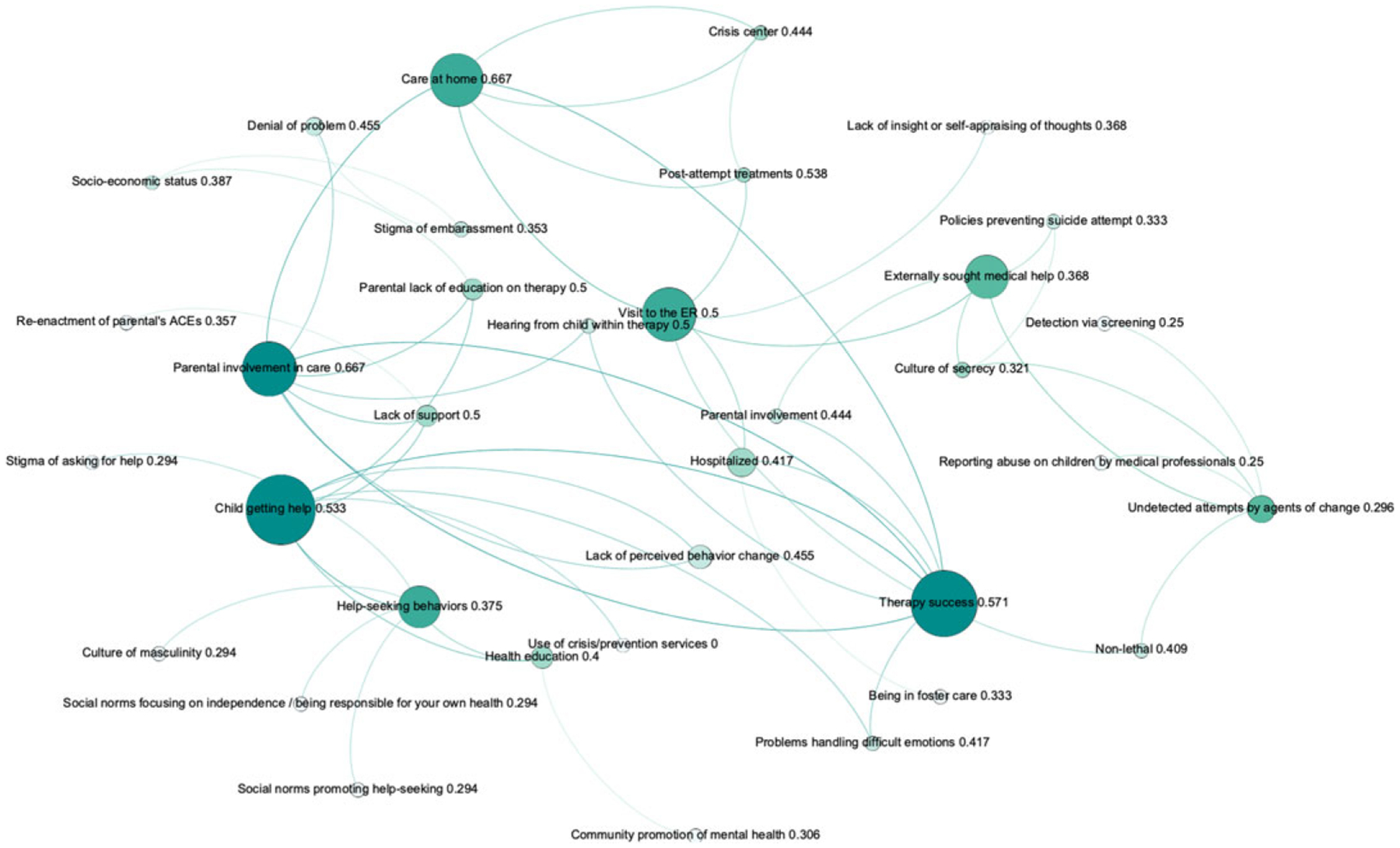
Fragment of the National Center for Injury Prevention and Control (NCIPC) model with three centralities: node size represents betweenness centrality (i.e., a larger node has control over a larger share of shortest paths), colors show degree centrality (nodes with more ties are darker), and the number indicates closeness centrality (which is based on distances). Betweenness centrality tracks how many times a concept would be traversed, hence we see that “child getting help” and “visit to the ER” are commonly occurring steps (i.e., frequently traversed) through the journey of an individual experiencing suicide ideation or attempts. Degree centrality reveals the extent to which a construct directly relates to another construct (e.g., therapy success is driven by parental involvement and also contributes to handling different emotions).

A network can be analyzed at different levels of granularity. McGlashan and colleagues have summarized how several network analyses can be used on a systems map, defining each analysis (e.g., density, degree) along its interpretation in the context of a systems map and the takeaway message for interventions (c.f. Table 1 in McGlashan et al. (2016)). The most granular analysis is performed at the level of individual nodes, using centrality measures to capture the “importance” of a node with respect to a desired property (e.g., degree, flow). Although node centrality is a well-known analytical method in network science, the interpretation of results is field-dependent. Table 1 lists the typical centrality measures used in systems maps together with their interpretation in the context of intervention planning for public health. Figure 2 exemplifies how centralities might be calculated on a fragment of the NCIPC model. These centrality measures have been used in numerous other studies on network science for public health (Power et al., 2022; Brauer et al., 2022; Smith et al., 2022), as well as in other comparative studies on systems maps (Krabbe, 2014; Kim and McCarthy, 2021; Wang and Giabbanelli, 2023).

At an intermediate level of granularity, we can analyze *groups* of nodes. This can be achieved by examining very local groups, through the *clustering coefficient*. For a given node, this measure quantifies the density of connections among the nodes’ neighbors. For example, if a node has four neighbors and they have three edges (out of 4 × 3 = 12 possible edges among four nodes), then the node has a clustering coefficient of $\frac{3}{12}=0.25$. The clustering coefficient of a network is obtained by averaging the clustering coefficient of individual nodes. While the *clustering coefficient* is a familiar construct in the literature on suicide, it is more commonly employed in neurophysiology studies through brain networks (Kim et al., 2024; Qin et al., 2024) or in computational social science via online social networks (Masuda et al., 2013; Colombo et al., 2016). In this paper, we use the clustering coefficient to study a network model of suicide. Larger groups of nodes can be studied through *communities*. Communities exist in certain subgroups where connections within the group are denser than connections to other groups. Communities can be useful for revealing hidden relationships among nodes (Lee and Lee, 2013), identifying themes (Chunaev, 2020), and visualization purposes (Fan, 2020). For example, communities can simplify a large system map into five or six themes, which become priority areas for prevention. Communities can serve to create “derivative maps” which are reduced to their groups, as illustrated by analyses of the Foresight Obesity Map which was reduced into a series of such derivative maps to guide policies (Finegood et al., 2010).

**Figure 3.**
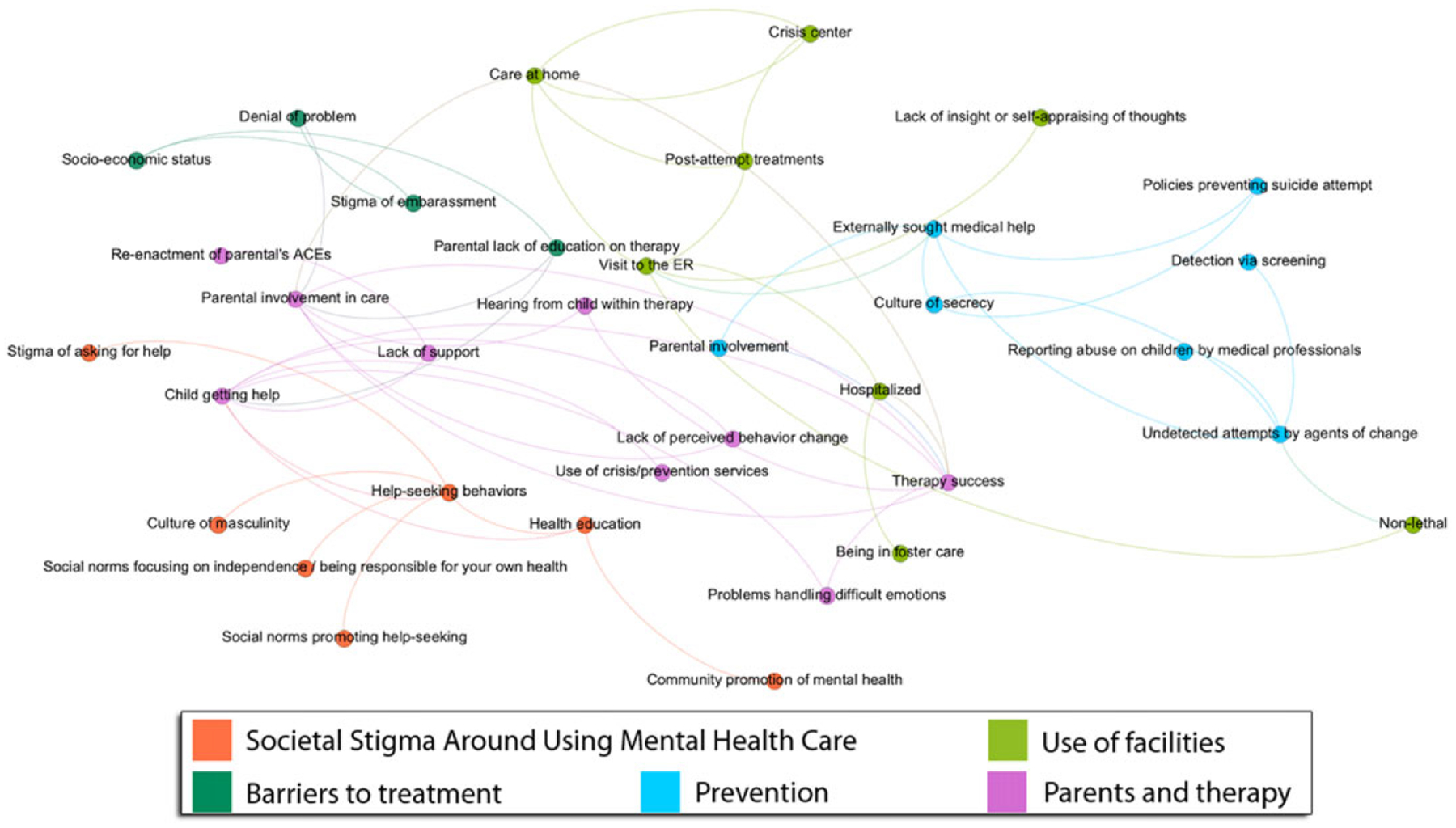
The Louvain method detected five communities in this fragment of the National Center for Injury Prevention and Control (NCIPC) model causal map, shown with distinct colors. Labels are only indicative, since the algorithm assigns concepts to communities but does not automatically name these communities.

The goal of community detection via algorithms is to divide nodes in such a way that there are many edges in subgroups and few edges between them (Newman, 2018). Multiple community detection algorithms have been developed (Clauset et al., 2004; Lancichinetti and Fortunato, 2009; Newman and Girvan, 2004), and we use two algorithms to support the triangulation of results (i.e., ensure that findings are not an artifact of one specific method). The *Louvain Algorithm* for community detection greedily maximizes modularity (Newman, 2018, p. 511–512) by repeatedly applying two steps: joining individual nodes into groups to find the arrangement with the highest modularity (which measures the density of links within a group as compared to links going outside) and restructuring the network by aggregating communities into “super nodes”. This iterative process is performed until no increase of modularity is possible. Figure 3 exemplifies the application of this method onto the same section of the NCIPC model as shown in the previous figure for centrality. In the worst case, communities detected using the Louvain Algorithm can be badly connected or even disconnected. The *Leiden Algorithm* extends the Louvain Algorithm to guarantee well-connected communities by using partition refinement. This algorithm consists of three steps: 1) local moving of nodes, 2) refinement of the partition, and 3) aggregation of the network based on the refined partition (Traag et al., 2019). By refining communities, the algorithm has more room to identify high-quality partitions. The use of several community detection algorithms on a map helps to establish the stability of the communities, hence ensuring that they reflect fundamental properties of the empirical data instead of being an artifact of one specific method.

At the level of the *whole network*, four measures are commonly included (McGlashan et al., 2016; McGlashan, 2018). The *density* refers to the portion of relationships that exist compared to how many relationships are possible. Table 2 shows examples of several policy application areas and their corresponding map densities to show some common densities for these types of maps. We note that the density in this application area is generally very low (i.e., we have sparse graphs). This property can be important for other aspects of the analysis, such as guiding the choice of centrality measures (Freund and Giabbanelli, 2022). Second, *distributions* for the in- and out-degrees of all nodes provide some insight into the general structure of a model. Unlike other fields, the intent in systems maps for policy-making is not typically to fit a certain distribution (e.g., test for a power-law). Rather, the examination tends to focus on three categories of degrees (Eden et al., 1992; Alomia-Hinojosa et al., 2022; Konti and Damigos, 2018; Giles et al., 2007): nodes without incoming edges (i.e., *sources* or “transmitters” with in-degree 0), which often constitute parameters of the model; nodes without outgoing edges (i.e., *sinks* or “receivers” with out-degree 0), which can serve as outputs; and *hubs* (large in- or out-degree), which can be prime targets for intervention planning due to their high connectivity. The *Average Path Length* is defined as the average of the shortest paths between each pair of nodes. It relates to the amount of effort that is needed to spread change, on average. Finally, the *diameter* measures the furthest distance between two nodes of the graph. Intuitively, a larger diameter indicates that a systems map has a wide “span” by covering far-away topics. The diameter has been extensively studied across various types of systems maps Strautmane (2012); Maksimenkova et al. (2018); Barbrook-Johnson and Carrick (2022).

## 3. Methodology

### 3.1 Comparing network models: node- and network-level metrics (Q1)

A network model can be built for different objectives. Small-scale models often serve to estimate the (direct and indirect) effects of a small set of variables of interest onto a suicide-related construct. For example, Kranzler et al. (2016) used an 11-node model to examine how self-injury and emotion dysregulation contribute to suicide attempt and Tang et al. (2010) used a six-node model to estimate how mental health constructs (e.g., post-traumatic stress disorder) ultimately shape the risk of suicide. In contrast, detailed models intended for intervention planning and evaluation have a much larger number of constructs and associated edges. Owing to their level of details and the significant investment required to create them via participatory modeling efforts (Section 2.3), there are fewer such models. For each of the four models examined in this paper, we obtained a directed network either directly from a file provided by the authors in a repository or supplementary materials (NCIPC and Brenas et al. (2019)), or by manually re-creating all edges and nodes from the authors’ figures (Chung, 2016; Page et al., 2018). Using the Python library NetworkX (version 2.6.2), we extracted characteristics for each of these four models at the node- and network-level (Section 2.4). Network-level characteristics include the number of nodes, number of edges, density, average path length, and degree distribution. Node-level characteristics include five node centrality measures: degree, Katz, Betweenness, Load, and Closeness. For each network, we focused on the top centralities, hence nodes were ranked by centrality.

### 3.2 Comparing perspectives: model, prevention package, and prevailing theories (Q2)

The SEM is a common framework for ACEs and suicide prevention (Centers for Disease Control and Prevention’s National Center for Injury Prevention and Control, 2021; Cramer and Kapusta, 2017). This approach considers that suicide-related factors belong to four different levels: individual, relationship, community, or societal. Taking the example of (Shagle and Barber, 1995), suicidal ideation is shaped by personal coping strategies (individual), family and peers (relationship), the school environment (community), and religion (societal). The CDC offers a complementary perspective through its suicide prevention technical package of policy, programs, and practices (Stone et al., 2017). In the technical package, concepts are classified into seven prevention strategies and approaches (Stone et al., 2017, p. 12–44), with an associated “shorthand notation” introduced here: strengthen economic supports (“economic”), strengthen access and delivery of suicide care (“care”), create protective environments (“environments”), promote connectedness (“connectedness”), teach coping and problem-solving skills (“coping”), identify and support people at risk (“support”), and lessen harms and prevent future risk (“prevent”).

In this study, we categorized the 361 nodes in the NCIPC Model per the SEM framework. This alignment is a qualitative endeavor: given the content of a model (i.e., the nodes’ names/labels), we seek to identify which aspect of a prevention strategy is covered and which level of the SEM framework is mobilized. Different individuals may interpret a model differently, which is a classic problem in reusing models (Freund and Giabbanelli, 2021). This creates the risk that findings reflect the particular interpretation of a group rather than the inherent characteristics of a model. To mediate this risk, we employ multiple coders from different backgrounds. By analyzing where their classifications concur, we can identify reliable categories. Specifically, we assigned nodes to four coders comprising two SMEs (in either suicide and/or ACEs) and two lay persons. Note that the non-SMEs and SMEs were not included in the same categorization groups and were instead split because of the differences in experience and knowledge in the specific areas of suicide and ACEs. Each coder assigned categories to each of their assigned nodes. They could disagree on a category; for example, a person may have seen “culture of secrecy” as a matter of environments and prevention strategies, while another may have seen it as environments and coping strategies. Coding conflicts were identified and resolved with a discussion between the coders, who explained the reasons for their classification and arrived at a consensus. In this example, one possible outcome could be the final categories of environments and coping. It was also possible that a consensus led to adding a category that was used by neither coder initially. This process has been documented in our online supplementary materials, with a “Nodes Additions” table devoted to categories that emerged as a result of inter-coder discussions. For a given node, the inter-coder agreement is defined as the fraction of categories assigned to the node that were endorsed by both coders. In our guiding example, since coders only shared the “environments” category out of three categories used in total, the inter-coder agreement is $\frac{1}{3}$.

### 3.3 Utilizing a model to guide policy: themes and intervention-oriented measures (Q3)

Complex problems such as adolescent suicide are addressed by myriad of actors, from the school (e.g., role models and after-school programs) to the community (e.g., psychologists and psychiatrists). Systems maps in other fields such as obesity have shown that the responsibilities of each actor may not fully align with the characteristics of the problem, thus leading to aspects for which nobody was clearly responsible, or aspects with shared responsibilities without clear communication lines. Cross-sector partnerships and whole-of-society models have thus emerged as multi-stakeholder approaches to policy-making in such problems (Dubé et al., 2014; Bekker et al., 2017), thus shifting from an actor- and problem-centric perspective (e.g., nutritionists and general practitioners for obesity) to a solution-centered approach (e.g., on themes such as body image and mental well-being). The decomposition of a map into themes is thus a key step for this paradigm shift, enabled by communication detection algorithms. Using the Python Library CDlib (version 0.2.5), we performed community detection on the NCIPC model using both the Louvain algorithm and the Leiden algorithm and compared their results to minimize the risk that communities are an artifact of one specific method. Each community was then named by manual inspection of its content and compared to key categories for suicide prevention from both the CDC suicide prevention technical package of policy, programs, and practices (Stone et al., 2017) and SEM framework (Centers for Disease Control and Prevention’s National Center for Injury Prevention and Control, 2021) (Section 3.2). The identification of communities helps find themes and coordinate multiple stakeholders to take and monitor interventions. The next step in using a system map is to evaluate the potential effect of such interventions (Mkhitaryan et al., 2020). Using the Actionable Systems tool (Giabbanelli and Baniukiewicz, 2018) to navigate networks, we show that the NCIPC model can support three types of interventions:

1. The *multiple paths*, as an intervention on one factor (e.g., access to mental health treatment), may have several direct and indirect effects onto an evaluation target (e.g., suicide ideation). We identify all disjoint paths between an intervention node and an outcome node.
2. The *feedback loops*, which can either work against an intervention (e.g., by creating inertia in the system and locking in certain dynamics) or help to amplify its effects (e.g., by reinforcing the initial impetus created by an intervention). We list all simple cycles in the map and categorize them as reinforcing or balancing based on standard terminology in systems science.^2^
3. The *rippling consequences*, as an intervention in one part of the map seeks to affect another specific part but may inadvertently have effects elsewhere. Hence we perform a core-decomposition centered on an intervention node, to reveal the parts of the map that would be affected at different distances.

Identifying multiple paths, feedback loops, and rippling effects is essential to comprehensively assess the cost-effectiveness of an intervention and mitigate unintended consequences. These types of measurements are a systems science approach to network-level analysis, thus complementing classical network science metrics such an examination of the system’s dynamics.

## 4. Results

### 4.1 Comparing network models (Q1)

**Figure 4.**
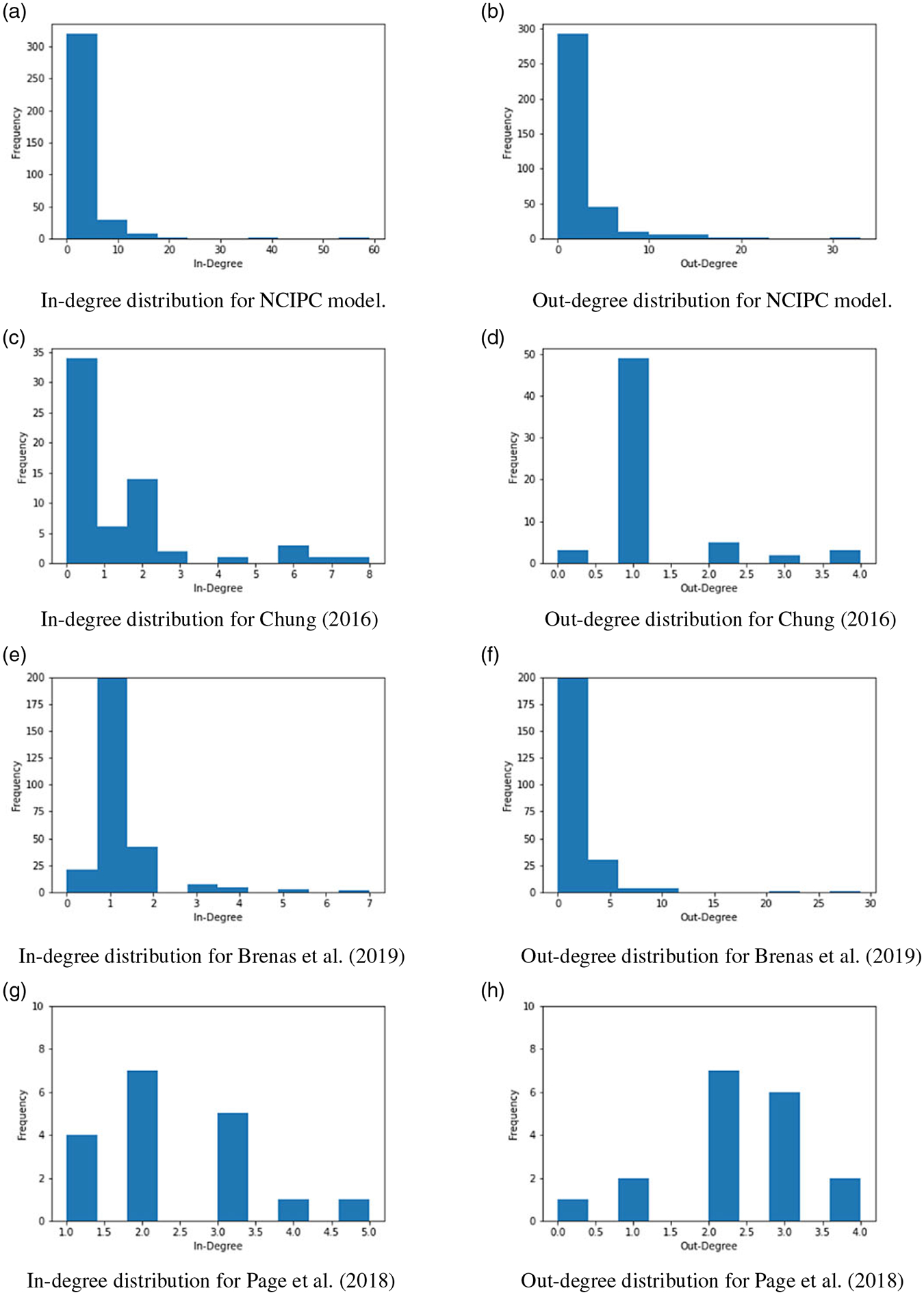
In-degree and out-degree distributions for four models. We note a skewed distribution indicating a high prevalence of low-degree nodes in the first three and a more uniform distribution in the last (bottom) model. The NCIPC model refers to the causal map created by the National Center for Injury Prevention and Control (NCIPC). Note that scales are different, since some models have fewer edges than others.

Network statistics for the four models are summarized in Table 3. We note that they are all sparse both globally (low density) and locally (low clustering), indicating that the SMEs were highly selective in identifying relationships between nodes. The relatively slow increase in the diameter and average path length compared with the increase in the number of nodes as the models get larger (from left to right in Table 3) suggests that experts increased granularity pertaining to certain facets of suicide (e.g., ACEs) instead of expanding onto new domains. For example, the NCIPC model has six times as many nodes as Chung (2016), but about the same average path length and only a small increase in diameter (from 11 to 13). For the two largest models, we see that the average path length is very short relative to the network size^3^ so it is relatively easy for one factor of the model to affect another. From a participatory modeling viewpoint, nodes are thus generally related, and participants did not embark on long tangents with aspects that had little to do with other factors of the model. From a policy implication viewpoint, it also means that the existing system surrounding suicide is tightly interconnected, which likely necessitates the joint use of several intervention points to deliver a system-wide change. We also note that clustering is almost twice as large in the NCIPC model compared to the previous sizeable models. This higher clustering coefficient suggests that there are more tightly connected neighborhoods of nodes, which represent areas of related concepts. The clustering coefficient is thus further evidence that SMEs have delved further into suicide-related notions instead of expanding into previously uncovered themes. Distributions for in- and out-degree (Figure 4) are highly skewed in all models but Page et al. (2018), which is the smallest one with 18 nodes. Once models become sizeable, they clearly have a few “hub nodes” that attract a lot of attention and a large number of nodes with few connections. The identification of these hub nodes is thus of particular interest to examine the focal points of the models and their policy implications.

Centrality measures help identify and compare important nodes across models. We identified important nodes for each model by observing the highly ranked nodes across centrality measures. For example, in the NCIPC Model, ACEs were ranked first according to degree, load, and betweenness centrality and third according to Katz and closeness centrality. This process led us to identify ACEs, mental health disorders, suicide ideation, suicide attempts, and substance abuse as driving nodes in the NCIPC Model. Table 4 summarizes the top five nodes for all models with respect to five centrality measures (degree, betweenness, load, closeness, and Katz centrality); our online supplementary material includes tables listing up to the top ten nodes. In other models, prominent factors are sleep and alcohol disorders, capability and desire for suicide as well as attempts, distress, and past attempts (Brenas et al., 2019; Chung, 2016; Page et al., 2018).

At a high level, the top five nodes suggest that central factors across models are proximal to the individual, rather than community or societal constructs. This can be expected for network models, as their objective is to elucidate suicide of individuals, hence all contributing factors eventually point to the individual level. At a detailed level, three of the models have similar key constructs (NCIPC, Page et al. (2018); Chung (2016)): mental health (e.g., through disorders or distress) and suicide (re-attempts). Brenas et al. (2019) have more unique focal points due to emphasis on ACEs rather than suicide. Note that key concepts in Brenas et al. (2019) are not absent in larger maps such as the NCIPC map; rather, they are relatively less important in the wider scope of the model. For example, substance abuse is among the top-10 nodes for closeness centrality in the NCIPC model, hence reflecting the notion of substance use and misuse in Brenas et al. (2019).

When seeking to use highly central nodes for intervention design, it is important to note that such nodes may be the *outcomes* (i.e., cannot be directly changed) of the intervention or the *leverage point* (i.e., the action through which we change the system). For instance, suicide ideation or attempts are the outcomes that we wish to change, but we cannot directly change them. Leverage points that will ultimately affect these target outcomes would include firearm restriction, as it will lower access to lethal means and eventually prevent attempts. In the NCIPC model, the top five most central nodes per degree centrality are ACEs, suicide ideation, suicide attempt, mental health disorders, and poverty. The first three are target outcomes, while the latter two are potential intervention points. Since mental health disorders are also among the top five constructs per betweenness, we see that mental health is heavily involved throughout the journey of an individual from trauma to ideation and attempts. The complementary measures of centrality thus suggest that intervening on mental health disorders could have a high immediate effect (per the degree) and even more rippling effects (per betweenness). Ultimately, a network analysis alone cannot determine whether a highly central node is an outcome or a leverage point: this interpretation must be made by a SME. Other examples of leverage points suggested by the network analysis and domain expertise include addressing poverty (which has a high degree in the NCIPC model) and other social determinants of health (high degree in Brenas et al. (2019)) or improving housing and treatment for mental health disorders (which have high load centrality in Brenas et al. (2019) and the NCIPC model).

### 4.2 Comparing perspectives (Q2)

**Figure 5.**
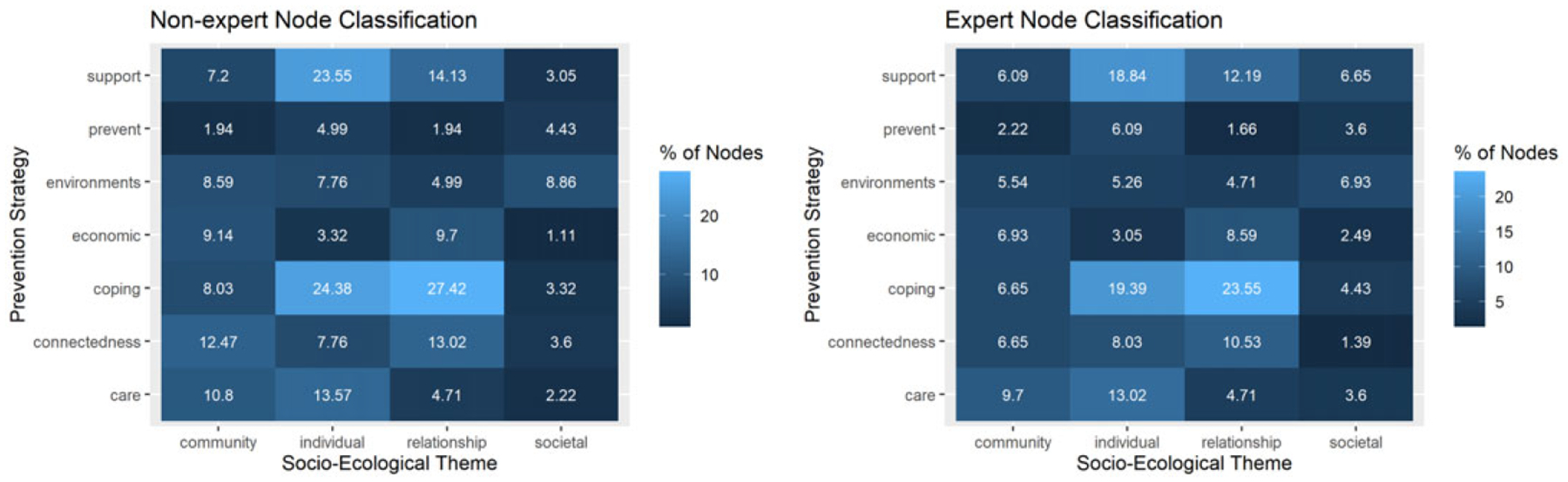
Percent of nodes assigned to each combination of prevention strategy and socio-ecological level. For example, 18.84% of nodes belong at the individual level, and the support prevention strategy could help address it according to the expert plot. Nodes can belong to multiple socio-ecological levels, and several prevention strategies may address them hence the total exceeds 100%.

Figure 5 summarizes how the nodes of the NCIPC map were categorized by lay individuals (left) and experts (right) with respect to both the SEM framework and the CDC suicide prevention technical package of policy, programs, and practices. Each group had two coders. Inter-coder agreement among lay individuals was 73.6% on the social-ecological level and 48.3% on the prevention strategy. During resolution, the lay coders realized that the CDC suicide prevention efforts were not mutually exclusive, but highly overlapping approaches that examine the problem from all sides.

Both lay coders and SMEs agreed that a large proportion of nodes fell into two major prevention strategies (teaching coping skills and identifying and supporting people at risk) and two social-ecological levels (individual and relationship). The intersection of relationship-level issues being solved by learning coping skills makes up 23.55% of all concepts covered in the NCIPC model. In both the SME and lay heatmaps (Figure 5), there is a significant decrease in the number of nodes affected by societal-level frameworks, making up at most 7% of all concepts in the NCIPC model. This suggests that many of the interventions that would impact adolescent suicide come from family, friends, and the community. We find the inclusion of the lay coders to be important, as it helps display gaps in public knowledge and can suggest tailored communication initiatives to further educate community members with tools for suicide prevention.

### 4.3 Utilizing a model to guide policy (Q3)

#### 4.3.1 Leveraging community identification to identify suicide themes

Table 5 summarizes the communities found by the Louvain and Leiden algorithms on the NCIPC model, and how they compare to categories examined in Q2 (social-ecological theme, CDC prevention category). For example, when looking at the Personal and Community Acceptance of Identity community in the Louvain detection algorithm, we can see where many of the CDC’s current approaches, namely promoting connectedness of the community and teaching coping skills to individuals, can be applied to this area. Both algorithms selected their own communities without any prior bias being imposed on them by the researchers; they found many of the same communities, with some differences in the bottom half of the table. The Louvain and Leiden algorithms respectively extracted eleven and ten communities of nodes from the NCIPC Model. Five communities are nearly identical, and two are similar between the two algorithms. The Leiden algorithm detected one community where discrimination (i.e., discriminatory hiring practices, segregated housing) was prominent that Louvain did not. Moreover, the Louvain algorithm discovered a community encompassing the societal stigma around using mental health care that Leiden did not.

**Figure 6.**
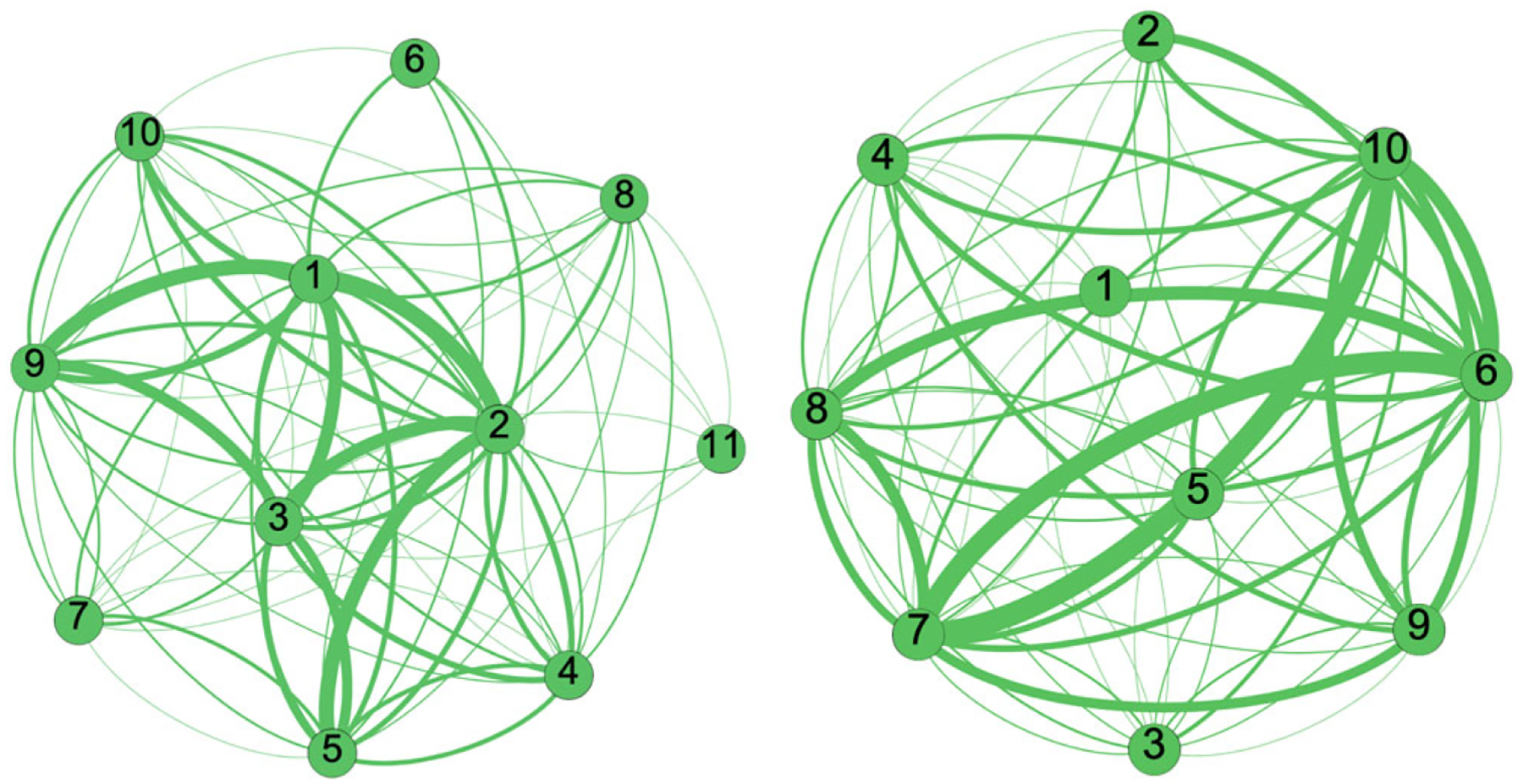
National Center for Injury Prevention and Control (NCIPC) model reduced based on the communities (comm.) detected by the Louvain (left) and Leiden algorithms (right). The adjacency matrices (top) provide a more compact view of the structures shown in node-and-link diagrams (bottom). Community numbers refer to Table 5.

Figure 6 displays the NCIPC Model reduced based on the communities detected by the Louvain and Leiden algorithms. The thickness of the arrow summarizes the strength of the connections between the communities based on the number of edges between their nodes. There are several points of agreement between the decompositions suggested by the two community detection algorithms. For example, ACEs (community #1) and access to health care and racial barriers (community #5) are strongly related to the rest of the system, which provides structural evidence that these communities form important themes for suicide prevention. However, the two algorithms offer widely different pictures on some themes, such as connectedness, suicide ideation, and attempt (community #6): it is weakly connected to anything with the Louvain algorithm (Figure 6; left) but one of the most highly connected themes per the Leiden algorithm (Figure 6; right). Suicide research would agree with the Leiden algorithm, as the vast majority of factors represented in the map are either protective or risk factors regarding suicide ideation and attempt, either directly or in relation with connectedness and ACEs. Furthermore, the Leiden algorithm correctly identifies the very strong relation coping skills and interpersonal relationships with peers (community #8) and ideation/attempt/connectedness, whereas the Louvain algorithm concludes that there is no such relation. This suggests that the Leiden algorithm is more apt to decompose our specific system into communities. Among the strong connections found by the Leiden Algorithm in Figure 6 (right), we note environmental factors on ACEs and development (community #7), socioeconomic factors, and discrimination (community #10). We also observe that household challenges and stress in the family (community #9) are involved in many other themes, such as emotional support from parents and crisis treatment (community #4) or socioeconomic factors and discrimination (community #10); these capture the impact of family stress onto youth and causes of family stress, respectively.

#### 4.3.2 Systems interventions

**Figure 7.**
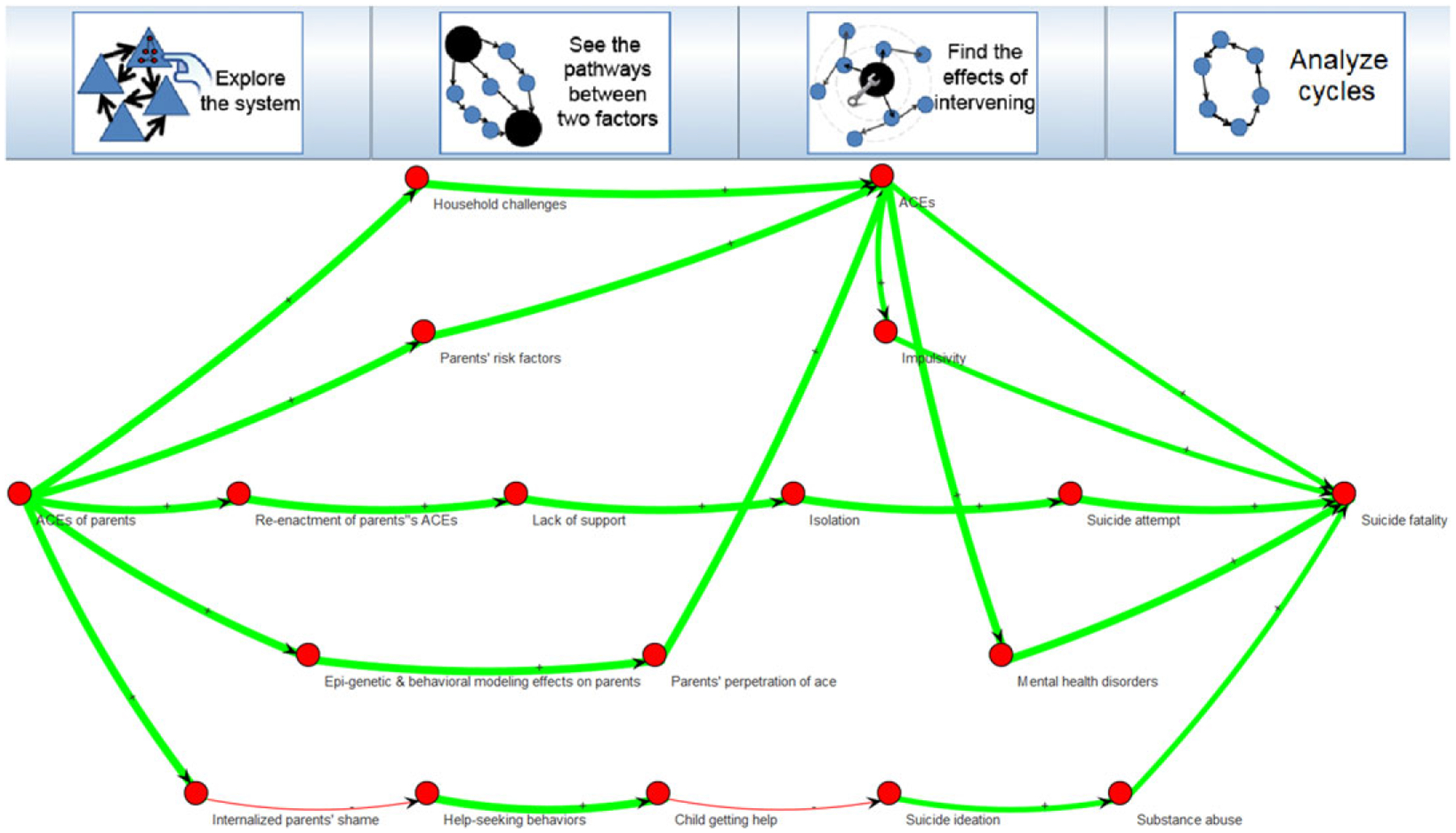
The ActionableSystems software allows users to automatically identify all pathways from one concept to another, as exemplified here by going from Adverse Childhood Experiences (ACEs) of parents (left) to suicide fatality (right).

We asked a SME on suicide and another SME on ACEs to provide queries that would commonly be used in their work, in order to illustrate each of the types of policy-oriented questions listed in Section 3.3. We start by investigating the notion of multiple paths between a construct of interest and a suicide outcome. Specifically, in Figure 7, we examined the many ways in which ACEs of parents eventually contribute to suicide fatality of the child, thus highlighting intergenerational effects. These effects are well established in the literature on suicide, as a recent review showed that nearly all studies demonstrated the effect of parental ACEs onto their children, either directly or indirectly (Zhang et al., 2023).

**Figure 8.**
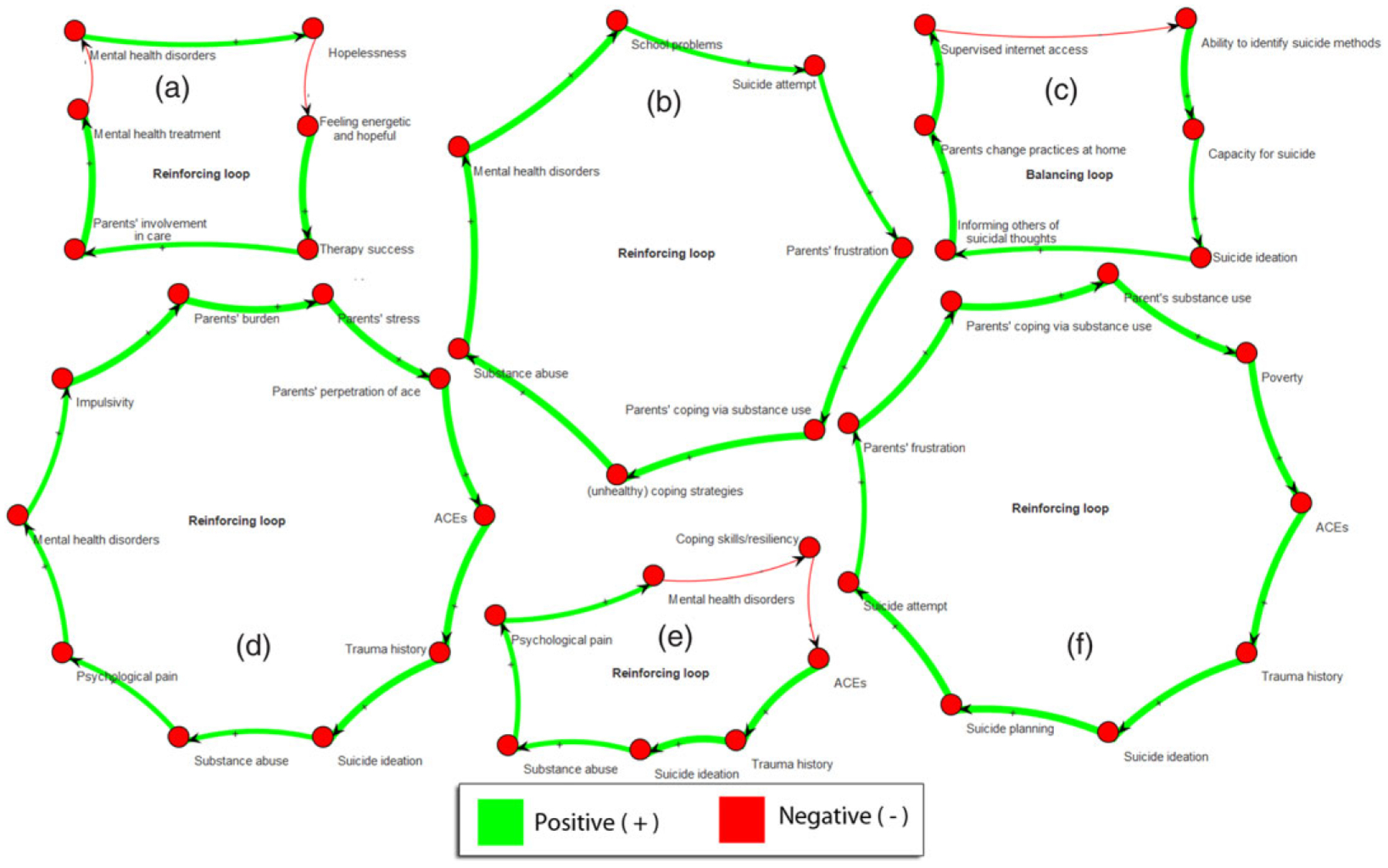
Sample loops from the map including (a) the benefits of therapy, (b) parental frustration and coping via substance use, (c) positive changes in practices that decrease capacity for suicide, parental perpetration of Adverse Childhood Experiences (ACEs), (e) mental health disorders, and (e) poverty.

Next, we exemplify the concept of feedback loops, whereby the effect of a factor ultimately impacts the same factor either in a reinforcing (i.e., amplifying) or balancing (i.e., dampening the dynamics) manner. In Figure 8, we selected a sample of six feedback loops covering different domains, from the benefits of therapy and changes in parental practices (Figure 8a/c) to the many loops involving parental frustration and coping via substance abuse or perpetration of ACEs (Figure 8b/d). We note that similar loops have been reported in other studies, such as loops between children’s well-being and parenting quality (Smith et al., 2021). Thanks to its broad coverage of proximal and distal factors, the loops also include high-level societal constructs such as poverty, thus demonstrating the perpetuation of social issues (Figure 8e). Loops involving poverty in suicide have also been reported in recent mapping studies, for instance as poverty creates more unmet physical, emotional, and social needs, which ultimately impair learning and create a barrier to career attainment, hence reinforcing poverty (Vu et al., 2022).

**Figure 9.**
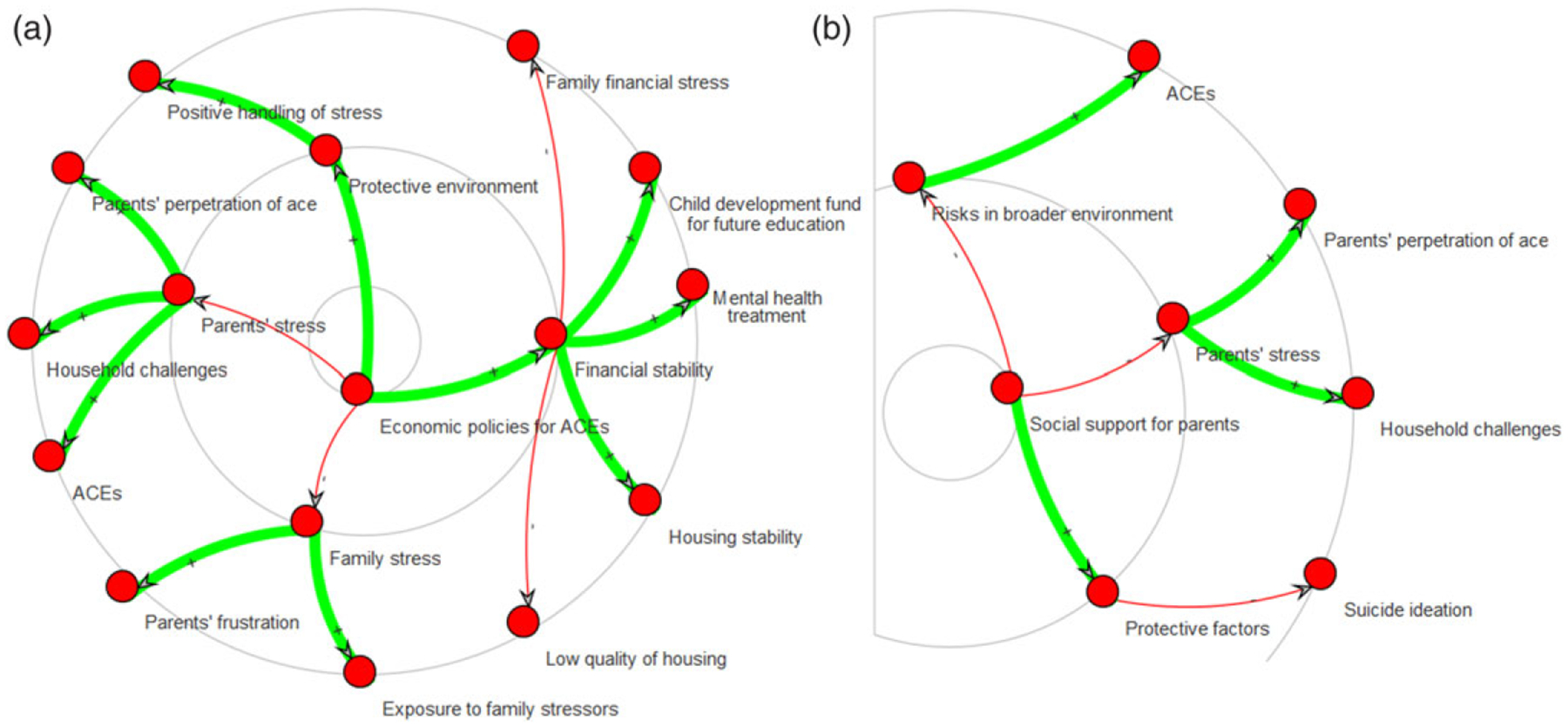
Rippling effects of interventions on economic policies (a) and social support for parents (partial view; b). Effects are shown in concentric circles from most proximal to more distal. Both sub-figures only depict a part of the map, as the user controls the maximum distance up to which effects should be identified.

Finally, we illustrate the idea of rippling effects, where an intervention targets a specific construct and its consequences are examined through the broader map. Economic policies (Figure 9) can directly improve financial stability and lower stress, which ultimately reduces ACEs. A systematic review of the evidence further supports that upstream socioeconomic interventions eventually lower ACEs, while noting that effect strengths are different depending on the ACE (e.g., modest for adverse parenting, strongest for childhood victimization) (Courtin et al., 2019). Social support for parents (Figure 9) also reduces ACEs, in addition to preventing suicide ideation. The protection that social support for parents confer against ACEs in their children has been discussed in several studies (Narayan et al., 2021).

## 5. Discussion

Research on the associations between ACEs and suicide has typically been limited due to methodological constraints. The development of a large-scale system map of ACEs and suicide (i.e., the NCIPC model) served as a catalyst for expanding research between these two factors. In this paper, we applied network analysis to examine this system map to compare it against other maps and to find useful patterns between ACEs and suicide (ideation, attempt, and fatality) for informing suicide prevention efforts. While network science has been used to examine systems maps in fields such as obesity, there has been limited research at the intersection of suicide and network science. This paper helps to address this gap. In comparing the NCIPC model to other maps, the NCIPC model stands out as more comprehensive in concepts and relationships. It contains concepts captured by the previous models, such as the topics of suicide treatment, mental disorders, and other key concepts from Page et al. (2018), and mental health disorders from Chung (2016). The reason this model is noticeably more comprehensive is its inclusion of community-level factors (e.g., poverty, violence), policy-level factors (e.g., healthcare policies, responsible suicide awareness education), and the family unit (e.g., ACEs of parents, parent substance abuse). In short, the NCIPC model broadens the view of suicide by shifting from a predominantly individual-centric view into a social-ecological framework.

Trauma is not just an individual trait or behavior. Trauma is perpetuated through familial, social, and historical context. In the NCIPC model linking ACEs and suicide risk, families and parents appear to be an important contributor to the intergenerational transmission of ACEs, as evidenced in Figures 8 and 9 where both parental mental health and financial instability partially explain the relationship between exposure to ACEs and suicidal thoughts and behaviors. Thus, the map underscores the importance of trauma-informed interventions across all levels of the social ecology, and the importance of taking an intergenerational approach to addressing social determinants of health such as poverty, housing instability, and social disadvantage. Policies that increase access to mental health services (particularly for parents) and economic supports for families are critical for interrupting the intergenerational transmission of ACEs and suicide risk.

Our network analysis of the NCIPC model helps us understand the complex interactions that result in adolescent suicidal behavior. Given its large size, the NCIPC model is not meant to be visualized all at once. Rather, it provides a decision-support tool that can be utilized to address specific questions and to help identify direct and indirect risk and protective factors related to adolescent suicide.

There are two main technical limitations to this study. First, despite the recent increase in models for suicide prevention (Schuerkamp et al., 2023), there are relatively few comprehensive systems maps. The comparative portion of our analysis was thus limited to the number of models available. We note that new models have emerged since our study was undertaken (Vu et al., 2022), and there are also several new systems maps on sub-systems involved in suicide and ACEs such as homelessness (OBrien et al., 2024) and substance abuse (Smith et al., 2021). Second, there is a seemingly endless number of measures that could be used to characterize networks at the level of individual elements, groups, or for the whole network. For instance, other studies have reported the “ruggedness” (i.e., number of components) (Strautmane, 2012), the number of paths (Ifenthaler, 2010), or custom measures (e.g., percentage of nodes with in- and out-degree larger than one in Plate (2010)). It was recently shown that many of these measures are related, hence using more measures does not necessarily yield more insight (Wang and Giabbanelli, 2023). We thus focused on measures that are commonly used in systems science, so that our findings can be interpreted within the context of public health.

The goal of a model is not to re-create the complexity of a real-world problem. Rather, a model is necessarily a *simplification*, which provides guidance to identify and evaluate solutions. Continuously developing larger models or integrating existing ones into a form of “super-model” is thus likely to produce an overly complex model, as discussed in (Voinov and Shugart, 2013). Since we showed that there are already several high-quality models for suicide and ACEs, future work could focus on the transition from comprehensive high-level qualitative models onto perhaps smaller but more data-driven and quantitative models. Such models would provide better support for systems intervention, as they would be able to measure the strength of an effect or when it would occur, in contrast to current qualitative models that can only list which factors would be impacted. Such a transition has recently started, as the content of the NCIPC model was contrasted with several national datasets, showing that some parts of the model could be measured (e.g., those closest to the individual) whereas other parts suffered from a lack of data (e.g., societal-level constructs) (Giabbanelli et al., 2023). Other studies have built on systems maps to offer comprehensive simulation models which can report the cost-effectiveness of interventions (Crosland et al., 2024). A recent comment observed that “planners are not opposed to the use of systems models when the outputs help answer questions that align with the fundamental preferences of the decision-making structures within which they work” (Rock et al., 2024). It is thus particularly promising to leverage the information structured in systems maps and use the available data to support smaller models that answer practical questions from policymakers, clinicians, or caregivers.

## 6. Conclusion

Our comparative study suggests that SMEs and modelers were highly selective in identifying relationships when creating systems maps for suicide and adverse childhood experiences. When models got larger, they tended to expand on existing themes (e.g., increased level of details on trauma) rather than add an entirely new aspect. The maps are usually constructed around suicide ideation and attempt (which are frequently among the most central factors), but other highly central factors include potential intervention points such as mental health disorders. The interventions most represented in the NCIPC model include teaching coping skills and identifying and supporting people at risk, with a focus on the individual and relationship levels of the social-ecological framework. Decomposing the NCIPC model into communities showed the strong relations between ACEs and access to health care and racial barriers.

## Supplementary Material

Supplement1

Supplement2

## Figures and Tables

**Table 1.** Node centrality measures and their interpretation in the context of systems maps on suicide

Index	Reference	Interpretation	Example
Degree	(Sabidussi, 1966)	Shows whether a factor will be directly influenced/will directly influence its neighbor concepts	An increase in average income in a community would directly influence crime rates, education, health, and numerous other factors
Katz	(Katz, 1953)	Shows how much a factor can affect all other factors on the map by computing its connectivity to other highly ranked nodes; note the influence of a node decreases over distance between concepts	A new company building a factory in a community might increase average income in a community, which would then indirectly affect the aforementioned factors such as crime rates
Betweenness	(Freeman, 1977)	Shows how important a node is to bridging adjacent concepts in terms of the raw number of shortest paths this concept is a part of; has a more local-level interpretation because it has no notion of how many total global short paths there are, so a concept with high centrality on this index might not have many graph-level bridging capabilities and might instead have good local-level bridging within a cluster of nodes	Suicide attempts would obviously be directly connected to suicide fatality, but they might bridge non-adjacent concepts, such as a person’s capability forsuicideand fearof death, and willingness to seek help and support from their family
Load	(Goh et al., 2001)	Similar to betweenness, this shows the bridging capabilities of a node based on the percentage of total short paths that pass through this node; a highly central node by this index will be a bridge for a higher percentage of pairwise paths between concepts that aren’t directly connected to one another, and thus this centrality is a much better indication of global centrality than betweenness	Same as above
Closeness	(Bavelas, 1950)	Shows how easily a concept can be accessed by other nodes on the graph (though this is skewed by nodes that are especially far from other nodes); this centrality can help determine how quickly a change in a network would be able to reach all other nodes in the network, as a highly central node minimizes the number of concepts it must “jump” to in order to affect other nodes	Changes in an individual’s mental health status are quite easily influenced by their economic situation as, for instance, poverty can affect them, but they also similarly influenced by unrelated factors such as how welcoming their church community is; additionally, since the distance between concepts is minimized, this mental health status concept can easily influence other nodessuch asACEs.

**Table 2.** Number of nodes, edges, density, and number of participants in participatory modeling studies that produced maps.

	#nodes	#edges	Density	#participants	Field of applications
(McGlashan et al., 2019)	114	209	0.016	61	Public health, Non-communicable disease
(Firmansyah et al., 2019)	52	98	0.037	10	Smart cities, Urban computing
(Shahvi et al., 2021)	26	88	0.257	5	Water governance
(Falcone and De Rosa, 2020)	21	124	0.276	10	Waste management
(Christen et al., 2015)	43	105	0.057	8	Agriculture
(Licheetal., 2014)	78	95	0.0156	23	Mergers and acquisitions

Note that most models have a low density (i.e., sparse graphs)

**Table 3.** Network measurements for all four network-based suicide models

	Page et al. (2018)	Chung (2016)	Brenas et al. (2019)	The NCIPC Model
Nodes	18	62	296	361
Edges	42	77	379	946
Average Clustering Coefficient	0.19	0.005	0.005	0.093
Average Path Length	3.796	5.461	3.946	5.668
Density	0.137	0.02	0.004	0.007
Diameter	7	11	11	13

**Table 4.** Some nodes have the same value for a centrality measure, indicated by multiple nodes with the same rank.

	Page et al. (2018)	Chung (2016)	Brenas etal. (2019)	The NCIPC Model
Degree	1: Known Distressed or active disorder 2: Waitingsecondary mhs 2: Black Hole 3: General Population 3: Known Suicide attempts	1: Increasing capability 2: Increasing desire 2: Change in self-worth 3: Attempts 4: Decreasing desire	1: ACES Scores 2: Social Determinant of Health 3: Disease or Disorder 3: Disease 3: disease1	1: ACEs 2: Suicide ideation 3: Suicide attempt 4: Mental health disorders 5: Poverty
Katz	1: Known Distressed or active disorder 2: Black Hole 3: Known Suicide attempts 4: Known Recovering splitter 5: Waitingsecondary mhs	1: Increasing capability 2: Change in self-worth 3: Attempts 4: Decreasing desire 5: Increasing desire	1: Sleep Disorder 1: Sleep disorder1 2: Alcohol Abuse1 2: Alcohol abuse 3: Mental disorder due to drug	1: Suicide ideation 2: Suicide attempt 3: ACEs 4: Mental health disorders 5: Suicide fatality
Betweenness	1: Known Distressed or active disorder 2: Known Suicide attempts 3: Known Recovering splitter 4: Post-attempt treat split 5: Black Hole	1: Attempts 2: Accumulating attempts 3: Increasing desire 4: Increasing capability 5: Capability for suicide	1: Alcohol Abuse1 1: Alcohol abuse 2: Sleep Disorder 2: Sleep disorder1 3: Disorders of initiating and maintaining sleep	1: ACEs 2: Suicide ideation 3: Substance abuse 4: Mental health disorders 5: Suicide attempt
Load	1: Known Distressed or active disorder 2: Known Suicide attempts 3: Post-attempt treat split 4: Known Recovering splitter 5: Black Hole	1: Attempts 2: Accumulating attempts 3: Increasing desire 4: Increasing capability 5: Capability for suicide	1: Housing, local environment and transport finding 2: Residence and accommodation circumstances—finding 3: Environmental finding 4: Social and personal history finding 5: Disposition	1: ACEs 2: Suicide ideation 3: Substance abuse 4: Mental health disorders 5: Suicide attempt
Closeness	1: Known Distressed or active disorder 2: Known Suicide attempts 3: Black Hole 4: Return to pop split 5: General Population	1: Decreasing desire 2: Attempts 3: Accumulating attempts 4: Increasing desire 5: Increasing capability	1: Alcohol Abuse1 1: Alcohol abuse 2: Sleep Disorder 2: Sleep disorder 1 3: Disorders of initiating and maintaining sleep	1: Suicide ideation 2: Suicide attempt 3: ACEs 4: Mental health disorders 5: Suicide fatality

Note that Brenas et al. (2019) have duplicated nodes which the above rankings reflect; Page et al. (2018) also have a special “Black Hole” node, which refers to an exit point where the relationships leave the scope of concepts in the model. Mental health is abbreviated as ‘mhs’ by the authors.

**Table 5.** Both community detection algorithms produced similar communities; entries are ordered such that these similar communities are placed next to each other for ease of visibility. The community numbers are also used in Figure 5

Louvain Algorithm				Leiden Algorithm			
Community Name	Social ecological Categories	Prevention Strategies	#Nodes	Community Name	Social ecological Categories	Prevention Strategies	#Nodes
①ACEs; underdeveloped understanding and cognition	Individual; relationship	Coping, support	9	①ACEs; underdeveloped understanding and cognition	Individual, relationship	Coping, support	9
② Personal and community acceptance of identity	Individual, relationship, community	Coping, connectedness	15	② Personal and community acceptance of identity	Individual, relationship, community	Coping, connectedness	28
③ Familial Stress with a Focus on Parent Conflict	Relationship	Coping, support	17	③ Familial stress with a Focus on parent conflict	Relationship	Coping, support	18
④ Emotional Support from Parents; Use of CrisisTreatment	Relationship, community	Coping, care, support	34	④ Emotional support from parents; use of crisis treatment	Relationship, community	Coping, care, support	35
⑤Accessto Health Care; Focus on Racial Barriers	Community	Care, economic	21	⑤Access to Health Care; Focus on Racial Barriers	Community	Care, economic	27
⑥Suicide Ideation and Attempts, Access to Means; Isolation	Individual, community societal	Coping, connectedness, environment, prevention, support	58	⑥Suicide Ideation and Attempts; Connectedness/Isolation; Access to Means	Individual, relationship, community, societal	Coping, support, prevent environments, connectedness, care	65
⑦ ACEs Effect on Development	Individual, relationship	Coping, support	47	⑦ ACEsand Environmental Factors Affecting Development	Individual, relationship	Coping, support	53
⑧ Coping Skills; Interpersonal Relationships with Peers	Individual, relationship, community	Coping, connectedness	42	⑧ Coping and Using Mental Health Services	Individual, community	Coping, care, support economic	33
⑨ Parental and Community Socioeconomic Issues	Relationship, community	Coping, economic	32	⑨ Household Challenges and Familial Stress	Relationship	Coping, connectedness, support, economic	31
⑩ Societal Stigma Around Using Mental Health Care	Individual, community, societal	Care, connectedness, coping, support	22	⑩ Socioeconomic Factors; Focus on Discrimination	Individual, relationship, community	Environment, coping, connectedness, economic	62
⑪ Access to Care; Socioeconomic Status; Community Connectedness	Relationship, community	Economic, care, connectedness	64				

## Data Availability

Our study is a secondary analysis of network data. The NCIPC model was released by its authors on a third-party repository by the Open Science Framework (OSF), which can be publicly accessed without registration at https://osf.io/7nxp4/

## Footnotes

1 Systems Maps are similar to, yet distinct from, the directed acyclic graphs (DAGs) often found in the field of epidemiology. A system map *can* exhibit feedback loops, while DAGs cannot. Consequently, the assessment of cycles is central to the analysis of a systems map, but it cannot be done in DAGs.

2 In a causal map, each edge is categorized as “+” or “–” if its increase results in either an increase or decrease of a target, respectively. The category of a cycle depends on the category of its constituting edges. The effects of the edges multiply, hence two “−” cancel out and result in an increasing dynamic. Consequently, a cycle is “reinforcing” if it has none or an even number of “−” edges. Conversely, a cycle is “balancing” if it has an odd number of “−” edges. Being reinforcing or balancing does not suffice to know whether a cycle is “good” for an intervention, as it still depends on the content of a cycle. A cycle that reinforces suicidal ideation would be a problem, but a cycle that reinforces mental health treatment would be a benefit.

3 The average path length is less than the natural log of the number of nodes: *ln*(361) = 5.88 > 5.668 in the NCIPC model, *ln*(296) = 5.69 > 3.94 in Brenas et al. (2019). This qualifies as “short”.

## Appendix A: Brief summary of the NCIPC model

The NCIPC suicide map totals 361 suicide-related risk and protective factors related to adolescent suicide, stratified across four levels of the social ecology: individual, relationship, community, and societal. Each level provides risk and protective factors that were identified by at least one subject matter expert interviewed for constructing the map. Several factors may appear at more than one level given the interconnectedness of some risk and protective factors across the social ecology. At the individual level of the social ecology, our map identified variables that can be grouped into nine major categories. Personal identity includes beliefs that one’s identity is a personal choice, faith, identity as a sexual minority, and devaluation of one’s identity. Coping skills and resiliency cover constructs such as positive handling of stress, vulnerability, being equipped with psychological and interpersonal skills, unhealthy coping strategies, and tools for self-advocacy. Mental and emotional health is a prominent category, covering self-esteem, distress, psychological pain, self-destructive thoughts, feelings of self-worth, sense of fulfillment, feelings of insecurity, and interpersonal problems. Cognitive aptitudes are related to mental disability, brain development problems, and educational attainment. Physical health deals with chronic pain, co-morbidities, and physical disability. Impulsivity is an important trait, which includes acting in reaction to feelings, engaging in disruptive behaviors or risky behaviors. Factors in the map related to personal history include a history of ACEs or child maltreatment, prior exposure to violence, substance abuse, positive childhood experiences, historical trauma and its embedding in the family, previous suicide attempt, and experiences of failure. Experiences vary based on demographic and socioeconomic factors, including race and ethnicity, exposure to poverty, or homelessness. Finally, individuals have a different capacity for suicide, based on their access to lethal means, clarity of suicide planning (including the ability to identify suicide methods), and whether they feel capable to carry on suicide.

Moving one level away from the individual, the social ecology considers variables about relationships. For Adverse Childhood Experiences (ACEs), key relationships include parents (e.g., death/loss/separation from a parent, parents engage in illegal activities and are arrested or incarcerated, parent’s substance use or perpetration of ACEs) and the broader family unit (e.g., domestic violence, mental illness in the family). The family environment is characterized by many aspects, as supportive families can be beneficial (e.g., positive interactions with parents, nurturing environment, care at home, housing stability, cohesion of family) while dysfunctional house-holds are detrimental (e.g., parental escalation of conflict and harsh discipline, family history of secrecy, marital relationship stress, fighting in the house, parental lack of education on therapy). Relationships beyond the family units fall under the umbrella of peer relationships. Peers can positively influence a person’s life, for example by informing friends of suicidal intentions and thoughts, having supportive peers to talk to and receive social support or healthy mentoring. However, there are issues when peers are abusive (e.g., bullying), cope in unhealthy ways (e.g., substance use, risky behaviors), or do not provide adequate role models (e.g., peers in the justice system, suicides among friends).

The two highest levels of the social ecology are the community and society. At the community level of the social ecology our map identified variables that can be grouped into six major categories. First, there is access and availability of health services, particularly for mental health. Access and provision of healthcare services depend on the availability of related psychiatrists and the physical access. In a similar way, there is access and availability of social services, which provide education to get jobs as well as health education, quality childcare, and a crisis center. Characteristics of the neighborhood can be examined with respect to the environment (e.g., local jobs and promotion of mental health, or a segregated living with exposure to suicide) or safety (e.g., unsafe to go outside as neighborhood violence is normalized and regularly witnessed). The school environment plays a particular role for youth, as schools can be places that provide services and nurturing starting with good education in early childhood, or problematic environments that tolerate injustice and victimization such as labeling kids. The sixth category deals with community inequities, including discriminatory hiring practices, a history of segregation with the medical system, and population-level inequities. Finally, the societal-level encompasses cultural norms, policies, and structural inequities such as adversity and racism. Cultural norms can promote ACEs and impair treatment (e.g., stigmatizing ACEs or not seeing them as a problem, engaging in practices such as spanking, considering that one is responsible for their own health) or help individuals by promoting help-seeking behaviors and seeing suicide as unacceptable. Policies are far-ranging, as they include economic policies (e.g., health insurance), social policies (e.g., to increase connectedness), or reduce access to lethal means; these policies eventually prevent suicide attempt and completion.
